# Editorial Notes

**Published:** 1928

**Authors:** 


### Editorial Notes.

**Sir Dawson Williams, Editor of the British Medical Journal, 1898-1928.**

THE death of Sir Dawson Williams on February 27th cannot be allowed to pass unnoticed. He had resigned the Editorship of the *British Medical Journal* in December last, and instead of enjoying leisure at the end of his well-spent life, he died within a few weeks suddenly, but to himself not unexpectedly. His task as Editor of the *British Medical Journal* was not easy: the Journal has not only to publish articles which deal with the science and progress of medicine, it is the official chronicle of the proceedings of the Association and the organ to which every member turns to unburden himself of a message or a grievance. Dawson Williams used his great wisdom and rich experience wisely and generously. He would meet the criticism that papers of no scientific merit found place in the *British Medical Journal* by saying drily that he couldn't even be quite sure of the lasting value of the most scientific-looking communications, whilst the clinical experiences of the humblest practitioner might be useful. In reporting controversial discussions or in toning down embittered correspondence his influence was invaluable. Capable on occasions of forceful expression, he always tried to infuse dignity and generosity into controversy, for it was unavoidable that the Journal of the chief medical organisation of the Empire should sometimes deal with matters of dispute. Dawson Williams was well fitted to give written expression to the views, claims and hopes of the profession and the Association. In his hands the *British Medical Journal* set a high standard in periodical medical literature and avoided the pitfall of becoming the mere mouthpiece of a trade.

Some public tribute to his qualities as an editor is his due, but Dawson Williams had other gifts and charms which will make him sorely missed as a companion and a friend.

**The New Country Hospital at Winford.**

THE news that the Duke and Duchess of York have consented to lay the foundation stone of Bristol's new Country Hospital at Winford on July 7th has given great pleasure to those who have the interests of this project at heart. The immediate object of the hospital is to provide for the prolonged treatment, in a school-hospital, under the best conditions available, of chronic crippling disease in childhood. As this includes crippling heart disease, the hospital is brought into vital connection with the organisation for dealing with rheumatic heart disease which is growing up not in Bristol only, but also in the surrounding counties. For its support this hospital will depend mainly on grants—of which it is assured—from the Bristol Education Committee, and, it is hoped, from other local authorities whose children are treated there. Capital is still needed to provide for the building, as the Bristol Crippled Children's Society and the Bristol Orthopædic Hospital (who have pooled their interests for the sake of this conjoint undertaking) have thought it wise to buy a site large enough to prevent any likelihood of the hospital being built in, and also to allow of country annexes being added by the other Bristol hospitals as these become necessary. The possibility that this project might constitute an excellent form of war memorial for the city, which has been discussed, has much to be said in its favour, as much on the score of appropriateness as on that of utility, and it is to be hoped that it will be fully considered by the responsible authorities.

**The Royal Medico-Psychological Association.**

DURING February the Royal Medico-Psychological Association held a meeting in Bristol. At the dinner at the Royal Hotel on the 15th Professor G. M. Robertson spoke in terms of high praise of the University buildings, which the members of the Association had visited that afternoon, and expressed an earnest hope that the teaching of psychology might be taken up seriously and thoroughly in the Bristol School of Medicine.

Professor Nixon, in replying, assured the Association of the University's interest in the matter, pointing to the lectureship held by Dr. Barton White and the consultative psychiatric clinic presided over by Dr. Blachford at the General Hospital as evidences of this interest. On the following day the Association met at the Bristol Mental Hospital, Fishponds, and after a business session and lunch, at which the Lord Mayor of Bristol and the Registrar of the University were present, heard and discussed scientific papers.

**Conference on Rheumatic Diseases.**

A CONFERENCE on Rheumatic Diseases is to be held at Bath on Thursday and Friday, May 10th and 11th, 1928. Sir George Newman, Chief Medical Officer of the Ministry of Health, has kindly consented to act as President of the Conference. There will be three sessions : (1) Social

Aspects, presided over by Lord Dawson of Penn; (2) Causation, presided over by Sir Humphry Rolleston (Regius Professor of Physic, Cambridge); and (3) Treatment, presided over by Sir E. Farquhar Buzzard (Regius Professor of Medicine, Oxford). Many of the leading medical authorities have promised to contribute papers on the special phases of the subject upon which they are specially qualified to speak, and it is hoped that the Conference may be the means of throwing fresh light on some of its most pressing problems. The local Hon. medical secretary is Dr. Vincent Coates, 10 The Circus, Bath.

